# Identification of an inflammatory response-related gene prognostic signature and immune microenvironment for cervical cancer

**DOI:** 10.3389/fmolb.2024.1394902

**Published:** 2024-06-06

**Authors:** Zhuna Wu, Xuanxuan Zhuang, Meili Liang, Liying Sheng, Li Huang, Yanting Li, Yumin Ke

**Affiliations:** ^1^ Department of Gynecology and Obstetrics, The Second Affiliated Hospital of Fujian Medical University, Quanzhou, Fujian, China; ^2^ Department of Gynecology and Obstetrics, Anhai Hospital of Jinjiang, Quanzhou, Fujian, China

**Keywords:** cervical cancer, inflammation-related genes, predicted prognosis, immune microenvironment, drug sensitivity

## Abstract

**Background:** Cervical cancer (CC) is the fourth most common cancer among women worldwide. As part of the brisk cross-talk between the host and the tumor, prognosis can be affected through inflammatory responses or the tumor microenvironment. However, further exploration of the inflammatory response-related genes that have prognostic value, microenvironment infiltration, and chemotherapeutic therapies in CC is needed.

**Methods:** The clinical data and mRNA expression profiles of CC patients were downloaded from a public database for this study. In the TCGA cohort, a multigene prognostic signature was constructed by least absolute shrinkage and selection operator (LASSO) and Cox analyses. CC patients from the GEO cohort were used for validation. K‒M analysis was used to compare overall survival (OS) between the high- and low-risk groups. Univariate and multivariate Cox analyses were applied to determine the independent predictors of OS. The immune cell infiltration and immune-related functional score were calculated by single-sample gene set enrichment analysis (GSEA). Immunohistochemistry was utilized to validate the protein expression of prognostic genes in CC tissues.

**Results:** A genetic signature model associated with the inflammatory response was built by LASSO Cox regression analysis. Patients in the high-risk group had a significantly lower OS rate. The predictive ability of the prognostic genes was evaluated by means of receiver operating characteristic (ROC) curve analysis. The risk score was confirmed to be an independent predictor of OS by univariate and multivariate Cox analyses. The immune status differed between the high-risk and low-risk groups, and the cancer-related pathways were enriched in the high-risk group according to functional analysis. The risk score was significantly related to tumor stage and immune infiltration type. The expression levels of five prognostic genes (LCK, GCH1, TNFRSF9, ITGA5, and SLC7A1) were positively related to sensitivity to antitumor drugs. Additionally, the expression of prognostic genes was significantly different between CC tissues and myoma patient cervix (non-tumorous) tissues in the separate sample cohort.

**Conclusion:** A model consisting of 5 inflammation-related genes can be used to predict prognosis and influence immune status in CC patients. Furthermore, the inhibition or enhancement of these genes may become a novel alternative therapy.

## 1 Introduction

At present, CC is the most common malignant tumor of the female genital tract, with a high incidence at 40–59 years of age and a trend toward younger patients (Li et al., 2017; Sung et al., 2021). Globally, more than 500,000 women are diagnosed with CC, and at least 300,000 deaths are attributed to this disease every year (Cohen et al., 2019). Persistent infection with high-risk human papillomavirus (HPV) types 16 and 18 is a major factor in the development of cervical cancer. Cervical cancer and intraepithelial lesions are closely related to the activation of the inflammatory cascade and immune system (Yuan et al., 2021).

In recent years, an increasing number of studies have shown that inflammation can promote the occurrence and development of cancer (Armstrong et al., 2018; Nella Prevete et al., 2018; Sohrab et al., 2023). Tumor cells can promote the migration of many types of white blood cells to the tumor site by releasing a variety of cytokines and chemokines, including neutrophils, lymphocytes, macrophages, mast cells and dendritic cells. On the one hand, interferons, a key component of inflammation, which secreted by immune cells activate tumor-associated macrophages; on the other hand, they can promote cancer development by producing angiogenesis-related growth factors (Franciosi et al., 2022). Types of inflammation in cancer: different timing and inducers. Cancer-associated inflammation can precede carcinogenesis in form of autoimmunity or infection, can be induced by malignant cells or can be triggered by anti-cancer therapy (Greten and Grivennikov, 2019). Various cell intrinsic, host dependent or environmental factors can cause tumor-associated inflammation in different tumor types. Various Source/Stimulus: inactivation of tumor suppressors, loss of barrier function/commensal microorganisms, oncogene activation environment obesity and pollutants, carcinogenic microbes, hypoxia and cell death (Greten and Grivennikov, 2019). Persistent HPV infection allows the immune system to recognize it, which promotes different inflammatory responses that can contribute to the development of CC. Through cervicovaginal metabolic profiling, it can decipher the complex interplay between microbiota, HPV, inflammation and CC (Ilhan et al., 2019). Nevertheless, the association between genes related to the inflammatory response and the prognosis of CC patients remains unclear.

In this study, the mRNA expression profiles of CC patients and corresponding clinical data were downloaded from public databases. We then constructed prognostic biomarkers related to inflammatory responses for differentially expressed genes (DEGs) in the TCGA cohort and verified the reliability and stability of the model through the GEO cohort. We further performed functional enrichment analysis to explore the underlying mechanism involved. In addition, we analyzed the relationship between immune invasion type and prognostic gene expression. Furthermore, we investigated the relationship between the expression of prognostic genes related to tumor stemness and chemotherapy resistance. Finally, the differences in the expression of prognostic genes between CC and cervical fibroid (non-tumorous) tissues were verified via experiments.

## 2 Materials and methods

### 2.1 Collecting and processing microarray data (TCGA-CESC cohort and GEO-GSE44001 cohort)

Transcriptome RNA-seq data of 306 CESC cases and the matching clinical-pathological data were downloaded from the TCGA CESC dataset (https://portal.gdc.cancer.gov/repository). We obtained the matrix files of GSE44001 with another 300 CC samples from the Gene Expression Omnibus (GEO) database (https://www.ncbi.nlm.nih.gov/gds). Following the publication guidelines of TCGA and GEO and the data access policy, the data were published publicly from TCGA and GEO. Using the Molecular Signatures database, we identified 200 inflammatory response-related genes (Supplementary Table S1).

### 2.2 Construction and validation of genetic biomarkers associated with predictive inflammatory responses

DEGs between non-tumor tissues and tumor tissues in the TCGA cohort were identified by the “LIMMA” package with a |log fold change (FC)| > 1 and a false discovery rate (FDR) < 0.05. The prognostic value of inflammatory response-related genes was explored by univariate Cox analysis. To minimize the risk of overfitting, we built a prognostic model by conjugating LASSO (Simon et al., 2011) using the TCGA and GSE44001 datasets. In TCGA, the dependent variable was the status and overall survival of patients, and the independent variable in regression was the candidate prognostic DEGs. The risk score was calculated for the genes related to the inflammatory response and their regression coefficients. Patients were divided into low-risk and high-risk groups using the median risk score as the standard. In the constructed model, t-distributed stochastic neighbor embedding (t-SNE) analysis and principal component analysis (PCA) were performed with the “ggplot2” and “Rtsne” R packages to determine the distribution of different groups according to the expression levels of genes. The “survminer” R package was used to analyze OS among the high- and low-risk groups. The predictive value of prognostic genes was evaluated by time-dependent ROC curve analysis of the “time ROC” and “survival” R packages. In addition, the independent prognostic value of the 5 genes was explored by univariate and multivariate Cox analyses.

### 2.3 Functional enrichment analysis

To compare the DEGs between the low- and high-risk groups, gene set enrichment analysis (GSEA) was employed in Kyoto Encyclopedia of Genes and Genomes (KEGG) analyses. Among the high- and low-risk groups, the activities of 13 immune-related pathways and infiltration scores of 16 immune cells were computed by single-sample GSEA (ssGSEA).

### 2.4 Tumor microenvironment (TME) analysis

In different tumor tissues, the immune score and stromal score were used to analyze the infiltration degree of immune cells and stromal cells (Yoshihara et al., 2013). The relationship between those scores and the risk score was verified by Spearman correlation. Two-way ANOVA was utilized to test the associations between immune infiltration subtypes and risk scores. The Spearman correlation test was utilized to analyze the relationship between tumor stemness and the risk score.

### 2.5 Chemosensitivity analysis

The “Compound activity” and “RNA-seq” data were downloaded from the CellMiner interface (https://discover.nci.nih.gov/cellminer), the NCI-60 database, which includes 9 different types of tumors that contain 60 different cancer cell lines. Correlation analysis was performed for the efficacy of 314 drugs approved by the FDA (Supplementary Table S2). The relationship between drug sensitivity and prognostic gene expression was explored by Pearson correlation analysis.

### 2.6 Patient and tissue samples

Thirty-five paraffin-embedded CC specimens and thirty-three uterine normal cervical tissues were obtained at The Second Affiliated Hospital of Fujian Medical University (Fujian, China) from January 2018 to December 2018. The main treatment for all patients was hysterectomy resection. This research was approved by the Research Ethics Committee of The Second Affiliated Hospital of Fujian Medical University prior to the study.

### 2.7 Immunohistochemistry (IHC)

IHC staining was performed as previously described (Chen et al., 2016). The primary antibodies used were anti-GTP (Bioss, Beijing), anti-LCK (Immunoway, United States), anti-ITGA5 (Immunoway, United States) and anti-TNFRSF9 (Abmart, Shanghai). The proportions of GTP, LCK, ITGA5 and TNFRSF9 staining intensity were scored as follows: tan = 3; brownish yellow = 2; light yellow = 1; or negative = 0. The staining scope was scored as follows: more than 2/3 = 3; between 1/3 and 2/3 = 2; or less than 1/3 = 1. The final IHC scores for GTP, LCK, ITGA5 and TNFRSF9 expression were calculated by multiplying the two scores. The slides with scores of <3 or ≥3 were classified into high- and low-expression groups, respectively. In our study, the histopathological diagnosis was established by two pathologists specializing in gynecologic oncology.

### 2.8 Statistical analysis

We utilized R software to conduct (v.4.1.3) all the statistical analyses. We used the Wilcoxon test, chi-squared test, Mann–Whitney U test, Kaplan‒Meier test, univariate and multivariate Cox, Spearman or Pearson correlation analysis as described above. Differences with *p* < 0.05 were considered statistically significant for all the statistical analyses.

## 3 Result

### 3.1 Study procedure

**FIGURE 1:**
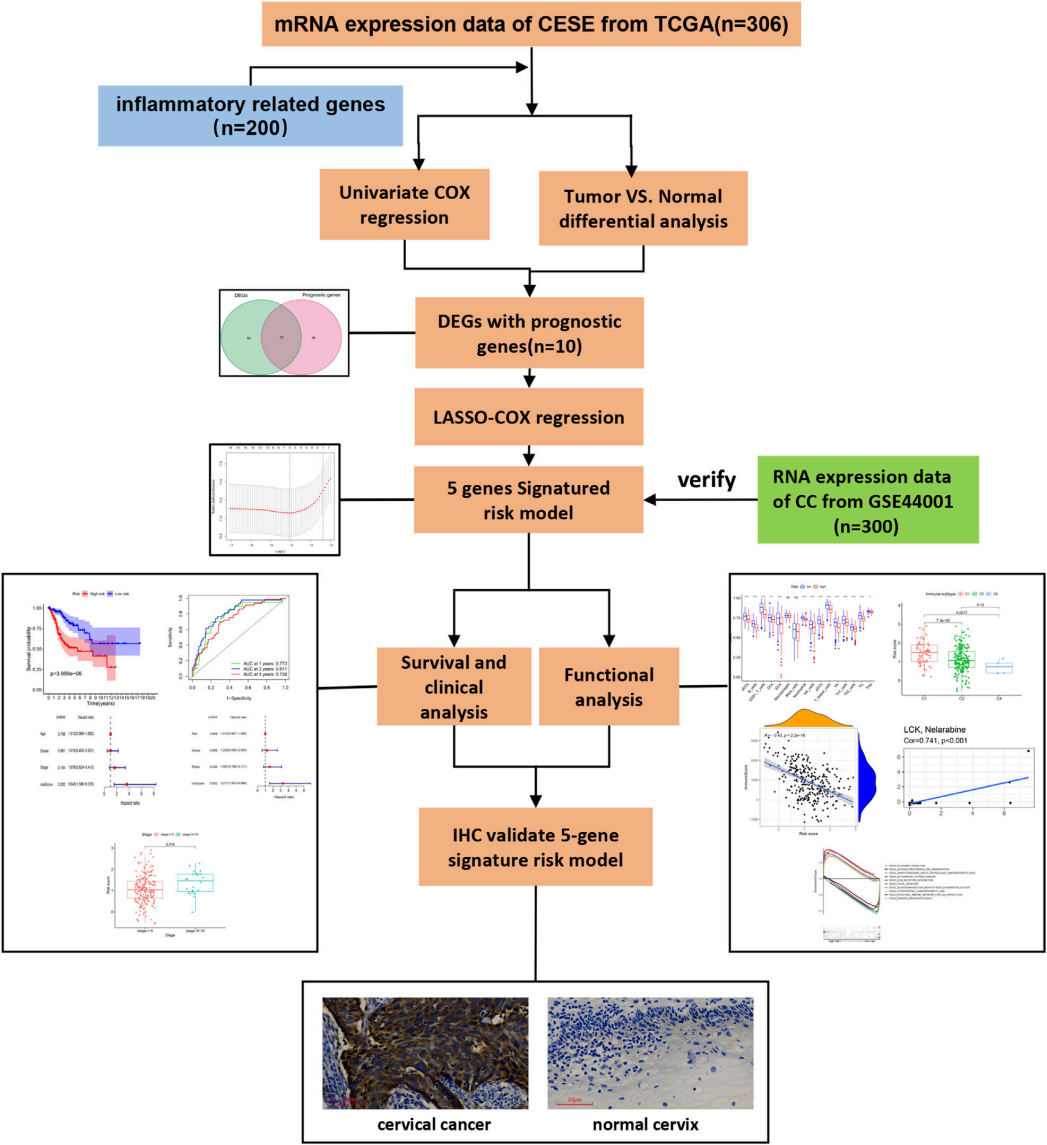
Analysis workflow of this study.

The analysis procedure used is shown in Figure 1. The transcriptome RNA-seq data were downloaded from the TCGA database. We identified DEGs with prognostic value between 306 CESE and inflammatory response-related genes. Next, LASSO-Cox regression was used to select the candidate genes, which composed a risk model, and then further verified in GSE44001. The risk model for further survival and functional analysis. Finally, the prognostic gene expression profile was verified by IHC.

### 3.2 Identification of inflammation-related DEGs in TCGA

**FIGURE 2:**
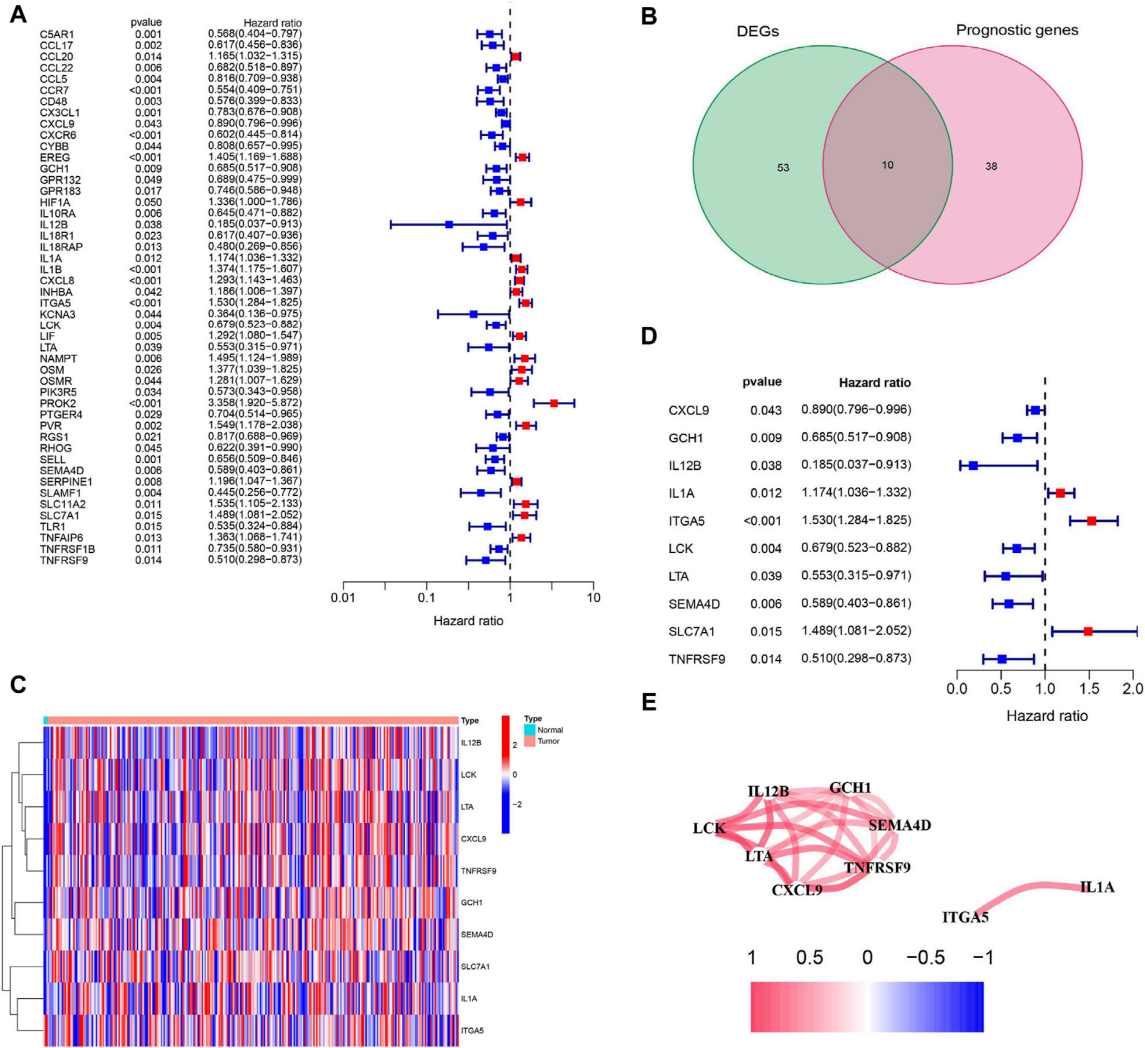
Identification of prognosis-related genes associated with inflammation response in the TCGA cohort. **(A)** Forest plots showing the results of prognosis-related genes by univariate cox analysis. High-risk genes represented by red bars, low-risk genes represented by blue bars. **(B)** Venn plot showing 10 prognosis-related DEGs shared between CC tissues and paracancerous tissues. **(C)** The heatmap plots showed that the 10 overlapping DEGs between CC tissues and paracancerous tissues. Column name of heatmap is the gene name, and row name is the ID of samples which not shown in plot. The colors from red to blue represent expression level from high to low in the heatmaps. **(D)** Forest plots showing the results of the association between 10 overlapping gene expression and OS. High-risk genes represented by red bars, low-risk genes represented by blue bars. **(E)** The correlation network of candidate genes. Red indicating positive correlation and blue indicating negative correlation. The darker the color, the more significant the correlation.

Sixty-three inflammation-related DEGs were identified between CC tissues and paracancerous normal tissues (Supplementary Table S3). Forty-eight genes related to prognosis were differentially expressed between tumor and paracancerous tissues (Figure 2A). Ten inflammation-related prognostic DEGs were identified between CC tissues and paracancerous normal tissues (Figures 2B, C). Univariate Cox analysis demonstrated that 10 of these genes were associated with OS, and the retention of 10 inflammatory response-related genes as prognostic indicators had a hazard ratio of 1.530 for ITGA5 (95% CI = 1.284–1.825, *p* < 0.001; Figure 2D). The correlations between these prognostic genes are shown in Figure 2E.

### 3.3 Construction and validation of a prognostic risk model in the TCGA cohort

**FIGURE 3:**
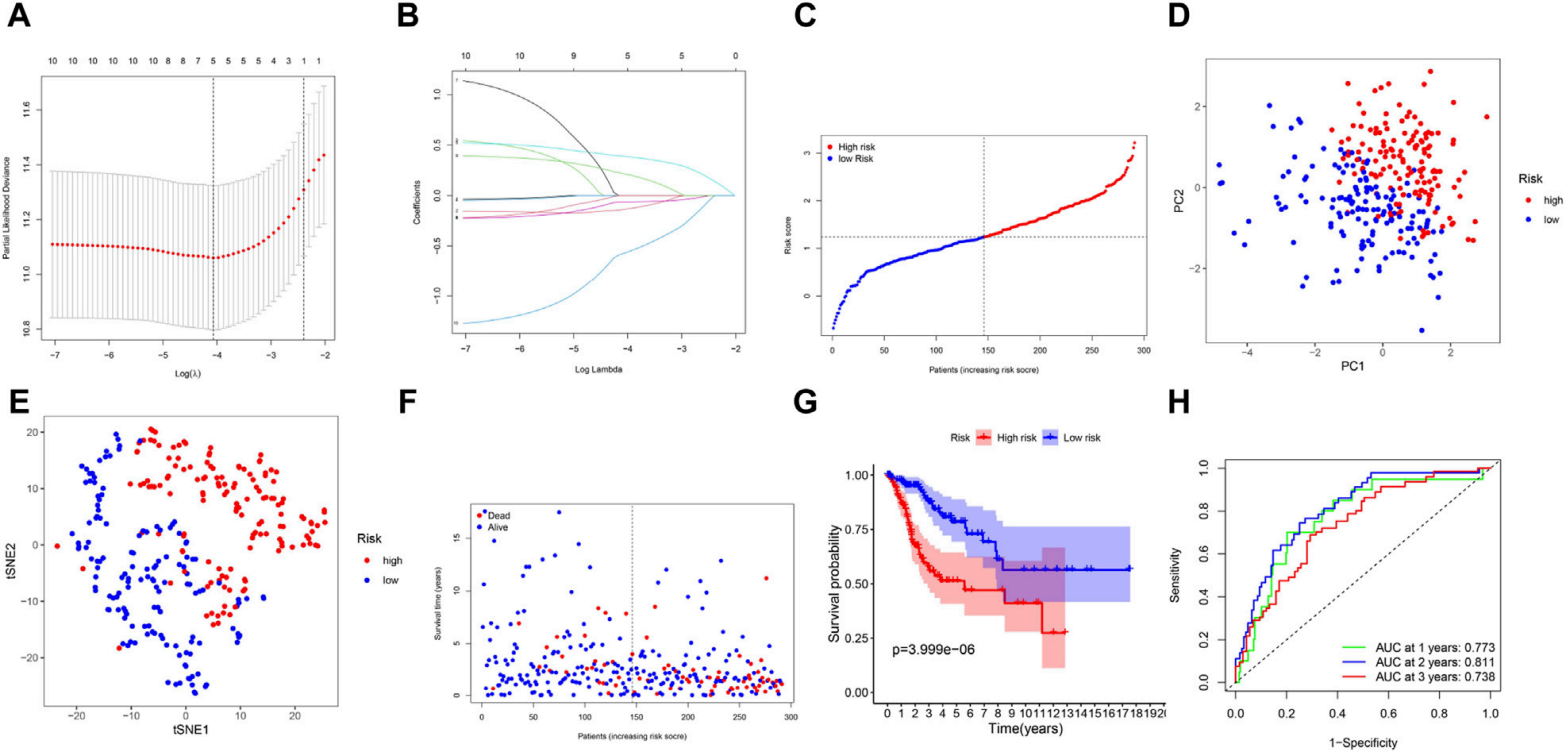
Prognostic analysis of the 5-gene signature model in the TCGA cohort. **(A)** Cross-validation in LASSO-Cox regression. **(B)** LASSO-Cox regression. **(C)** The distribution and median value of the risk scores. **(D)** PCA plot. **(E)** t-SNE analysis. **(F)** The distribution of OS status. **(G)** Kaplan-Meier curves for OS in the low- and high-risk groups. **(H)** AUC time-dependent ROC curves for OS.

By LASSO Cox regression analysis, we identified the expression profiles of the 10 genes mentioned above, and we generated a 5-gene prognostic model (Figure 3A). The prognostic risk score for each gene was calculated as follows: risk score = 0.389*expression level of ITGA5 + 0.217*expression level of SLC7A1-0.136*expression level of GCH1-0.063*expression level of LCK - 0.572*expression level of TNFRSF9 (Figure 3B). According to the median cutoff value, patients in the TCGA cohort were divided into two groups (Figure 3C). According to the PCA plot and t-SNE analysis, patients in different risk groups were separated in two different directions (Figures 3D, E). The scatter chart showed that patients at low risk were more likely to live longer than those at high risk (Figure 3F). In addition, the Kaplan‒Meier curve indicated that patients in the low-risk group had a significantly better OS than did those in the high-risk group (Figure 3G, *p* < 0.001). Then, we used time-dependent ROC curves to assess the prognostic ability of the risk score for survival prediction, and the area under the curve (AUC) reached 0.773 at 1 year, 0.811 at 2 years, and 0.738 at 3 years (Figure 3H).

### 3.4 Validation of the 5-gene signature in GSE44001

**FIGURE 4:**
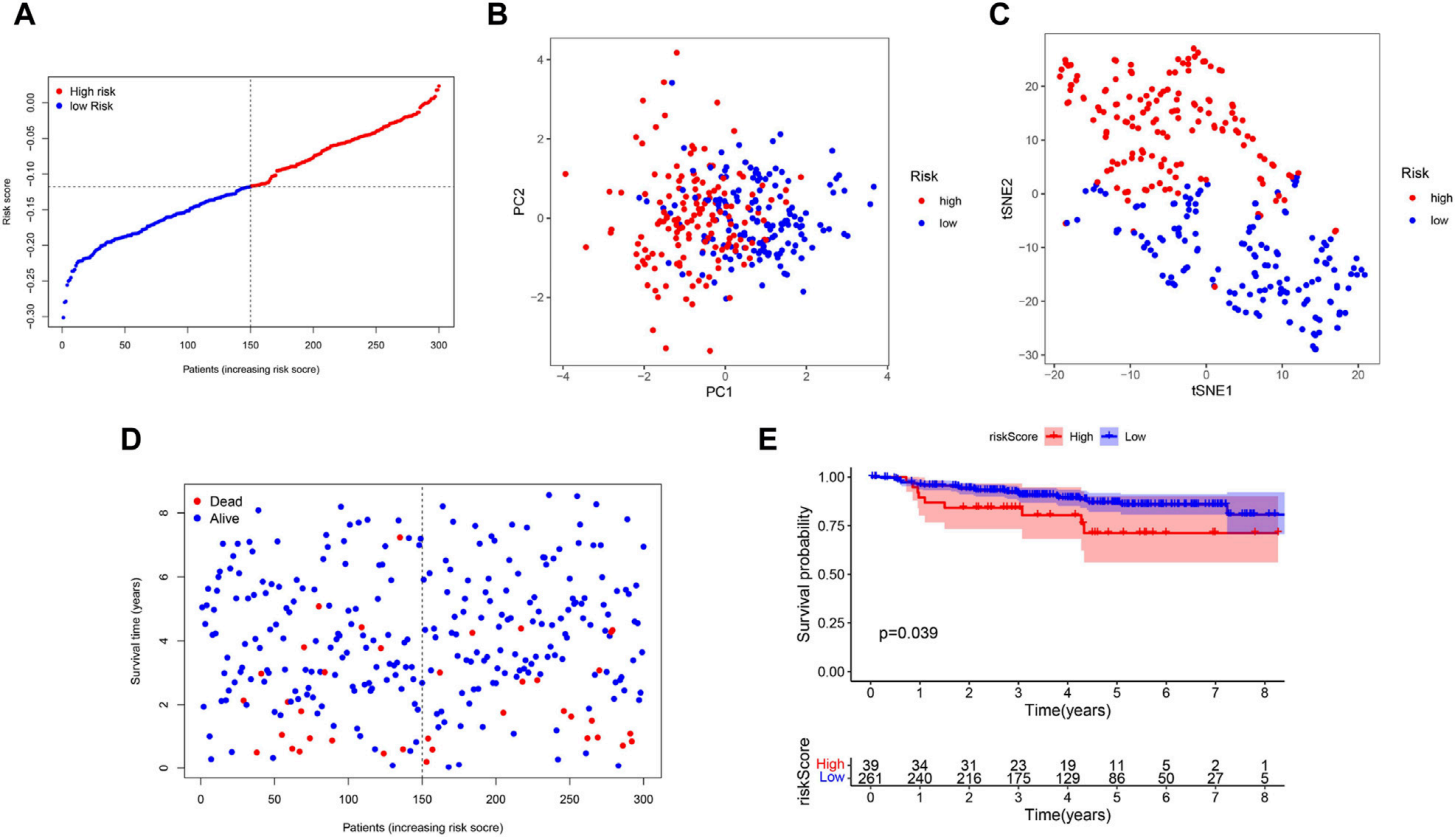
Validation of the 5-Gene Signature in the GSE44001, **(A)** The distribution and median value of the risk scores. **(B)** PCA plot. **(C)** t-SNE analysis. **(D)** The distribution of OS status. **(E)** Kaplan-Meier curves for OS in the low- and high-risk groups.

To verify the stability of the prognostic model built by the TCGA cohort, patients in GSE44001 were categorized into two groups based on the median value from the TCGA cohort: high-risk and low-risk groups (Figure 4A). The distribution of patients in the two subgroups was confirmed by PCA and t-distributed stochastic neighbor embedding (t-SNE) (Figures 4B, C). Similarly, patients in the low-risk group were more likely to live longer (Figure 4D) and have a longer survival time than were those in the high-risk group (Figure 4E).

### 3.5 Independent prognostic value and clinical features of the 5-gene signature risk score

**FIGURE 5:**
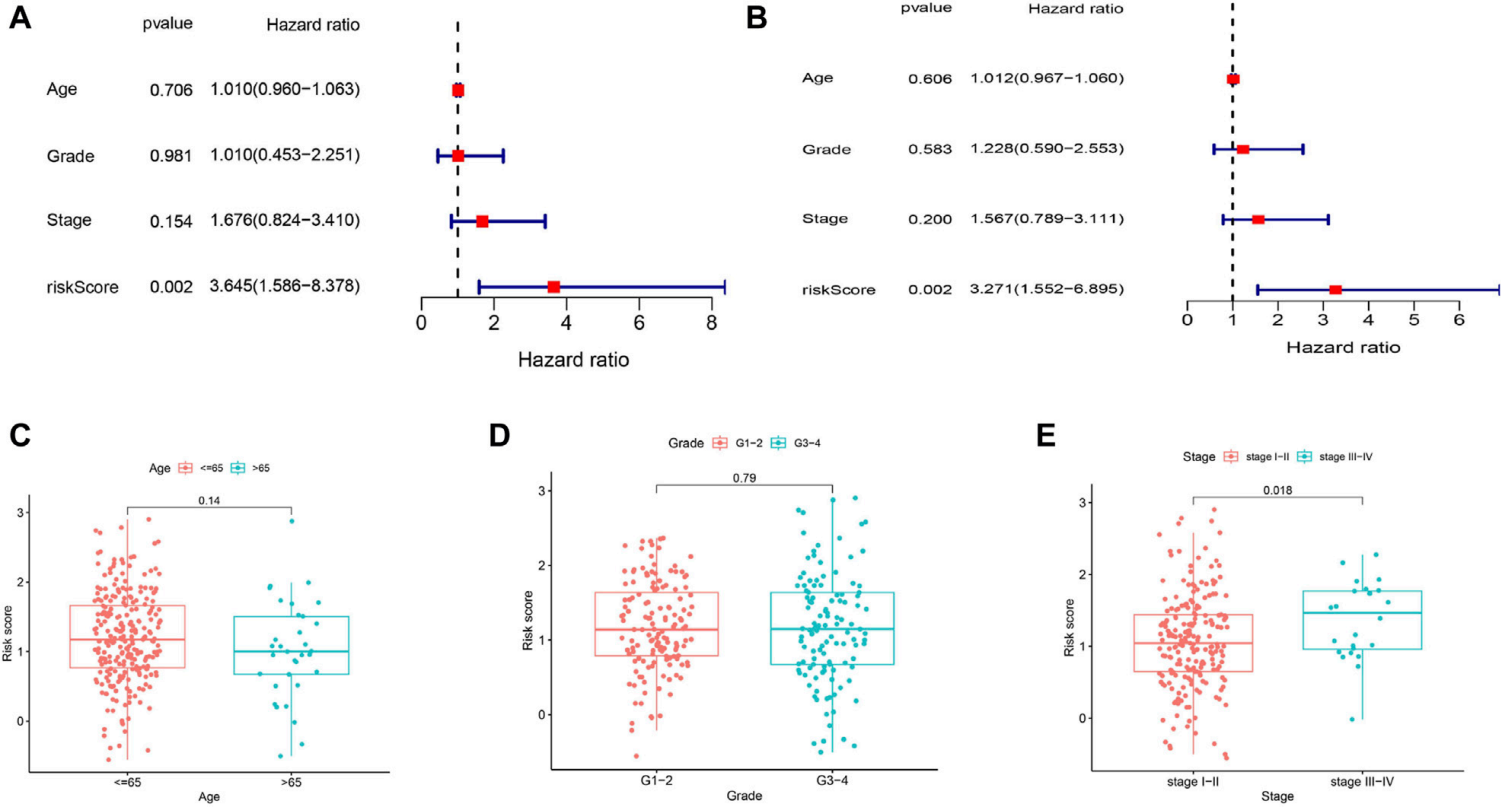
OS-related factors were screened, and the risk score divided by clinical characteristics in different groups. **(A)** Univariate Cox regression analyses screen OS-related factors. **(B)** Multivariate Cox regression analysis screen OS-related factors. **(C–E)** The risk score in different groups divided by clinical characteristics (chi-squared test). **(C)** Age. **(D)** Tumor Grade. **(E)** Tumor Stage.

To assess whether the risk score was an independent prognostic factor, we employed univariate and multivariate Cox analyses to analyze OS. According to the univariate Cox analysis, the risk score was significantly correlated with OS in the TCGA cohort (HR = 3.645, 95% CI = 1.586–8.378, *p* = 0.002) (Figure 5A). After correcting for other confounding variables, multivariate Cox analysis indicated that the risk score remained an independent predictor of OS (HR = 3.271, 95% CI = 1.552–6.895; *p* = 0.002) (Figure 5B). Analysis of the correlation between the risk score and the clinical characteristics of CC patients indicated that the risk score was not related to age or tumor grade (Figures 5C, D), and the risk score was significantly greater in patients with tumor stages III-IV (*p* < 0.05) than in those with tumor stages I-II (Figure 5E).

### 3.6 Analysis of immune status and the tumor microenvironment

**FIGURE 6:**
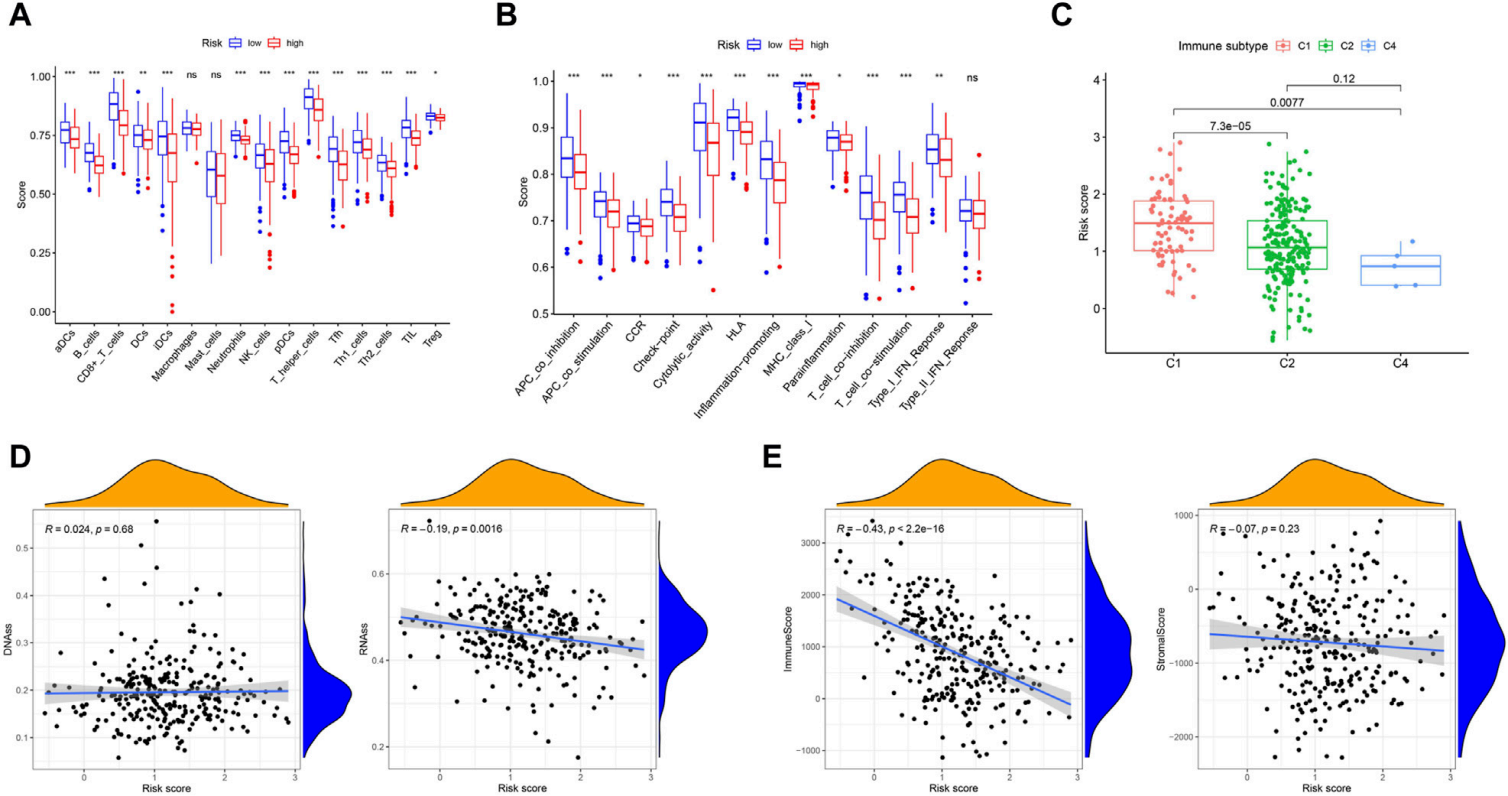
Immune status between different risk groups and the association between tumor microenvironment and risk score. **(A)** The scores of 16 immune cells were showed in boxplots by Wilcoxon text. **(B)** The 13 immune-related functions were showed in boxplots by Wilcoxon test. **(C)** Comparison of the risk score in different immune infiltration subtypes by Wilcoxon text. **(D)** The connection between risk score and DNAss, RNAss by Spearman correlation analysis. **(E)** The connection between risk score and ImmuneScore, StromalScore by Spearman correlation analysis. *p* values were showed as: ns, not significant; *p < 0.05; **p < 0.01; ***p < 0.001.

To further explore the relationship between immune status and the risk score, we quantified the enrichment scores of multiple immune cell subpopulations and related immune cell functions and pathways by ssGSEA. In the TCGA cohort, we detected that the levels of antigen-presenting genes, including aDCs, iDCs, pDCs, APC co-inhibition, APC co-stimulation, and HLA and MHC class I genes, significantly decreased in the high-risk group (all adjusted *p* < 0.05, Figures 6A, B).

To understand the association between the risk score and immune components, we tested the correlation between immune infiltrates and the risk score. In human solid tumors, six types of immune infiltrates, namely, wound healing (C1), INF-g dominant (C2), inflammatory (C3), lymphocyte depleted (C4), immunologically quiet (C5) and TGF-b dominant (C6), are recognized, which correspond to tumor promotion and tumor suppression, respectively (Tamborero et al., 2018a), No patient samples belonged to immune subtype Models C5 and C6 in CC. With respect to the TCGA-CESC data, we analyzed the immune infiltration of CC patients and correlated it with the risk score. The results indicated that a low risk score was significantly associated with C4, while a high risk score was significantly associated with C1 (Figure 6C). DNA stemness scores based on DNA methylation patterns (DNAss) and RNA stemness scores (RNAs) based on mRNA expression can be used to assess tumor stemness (Malta et al., 2018). The results showed that the risk score was significantly negatively correlated with RNAss (*p* = 0.0016) but not significantly associated with DNAss (Figure 6D). To estimate the tumor immune microenvironment, the immune score and stromal score were used. The results indicated that the risk score was significantly negatively correlated with the immune score (*p* < 0.001) but not significantly associated with the stromal score (Figure 6E).

**FIGURE 7:**
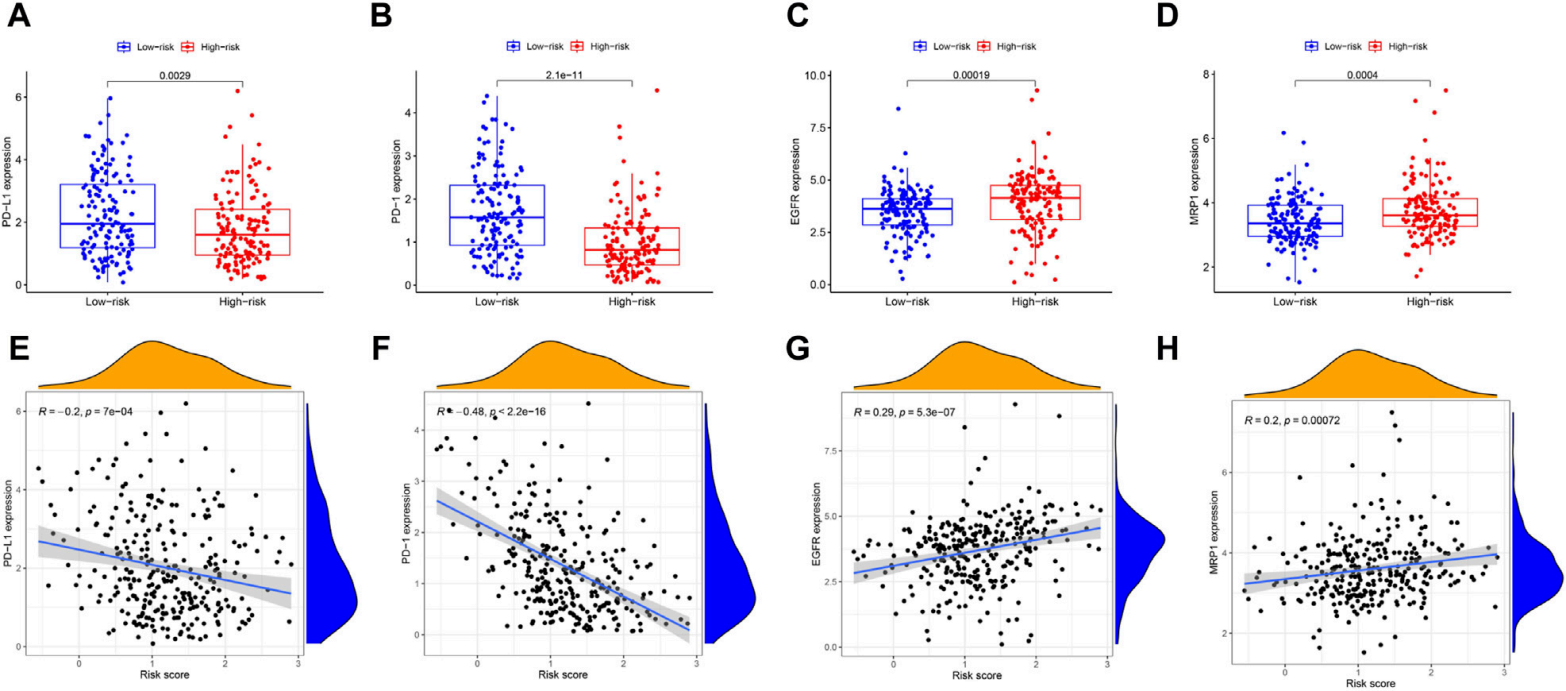
The comparison of the expression levels of PD-L1, PD-1, EGFR and MRP1 between different risk groups and association between the expression levels of PD-L1, PD-1, EGFR, MRP1 and risk score by spearman correlation analysis. **(A, E)** PD-L1. **(B, F)** PD-1. **(C, G)** EGFR. **(D, H)** MRP1.

In cancer immune evasion, PD-1/PD-L1 are key immune regulators. The expression level of PD-1/PD-L1 is a vital indicator for individualized targeted immunotherapy. The expression levels of PD-1/PD-L1 were significantly lower in the high-risk group than in the low-risk group (Figures 7A, B), and the expression level of PD-1/PD-L1 was negatively correlated with the risk score (Figures 7E, F). EGFR overexpression is associated with tumor metastasis, invasion and poor prognosis. The expression level of EGFR was significantly greater in the high-risk group than in the low-risk group (Figure 7C), and the expression level of EGFR was positively correlated with the risk score (Figure 7G). The MRP1 gene, which encodes a tumor drug resistance gene, was expressed at lower levels in the low-risk group than in the high-risk group (Figure 7D). Additionally, the expression of MRP1 was significantly positively related to the risk score (Figure 7H).

### 3.7 Pathway analysis

**FIGURE 8:**
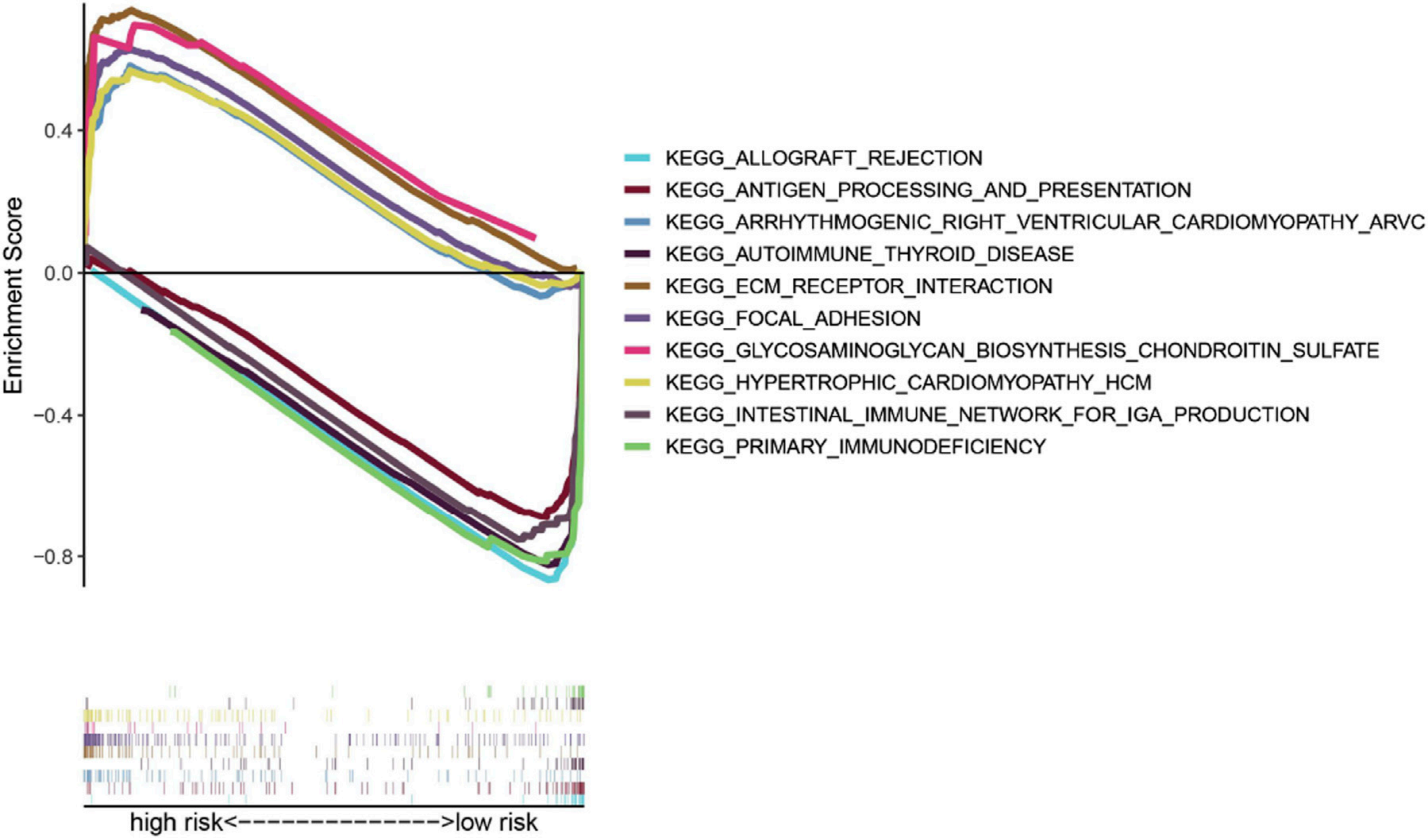
Gene set enrichment analysis of KEGG pathways.

To compare the low-risk and high-risk groups, we applied GSEA to perform KEGG pathway enrichment analysis. KEGG pathway enrichment analysis revealed that 5 KEGG pathways were enriched in the high-risk and low-risk groups with an FDR (false discovery rate) < 0.05 (Figure 8). The results revealed several pathways related to the inflammatory response, such as ECM receptor interaction, glycosaminoglycan biosynthesis-chondroitin sulfate, and hypertrophic cardiomyopathy (HCM). The KEGG pathways also included focal adhesion, which is correlated with cancer processes.

### 3.8 Prognosis-related genes expression and chemo-sensitivity of cancer cells

**FIGURE 9:**
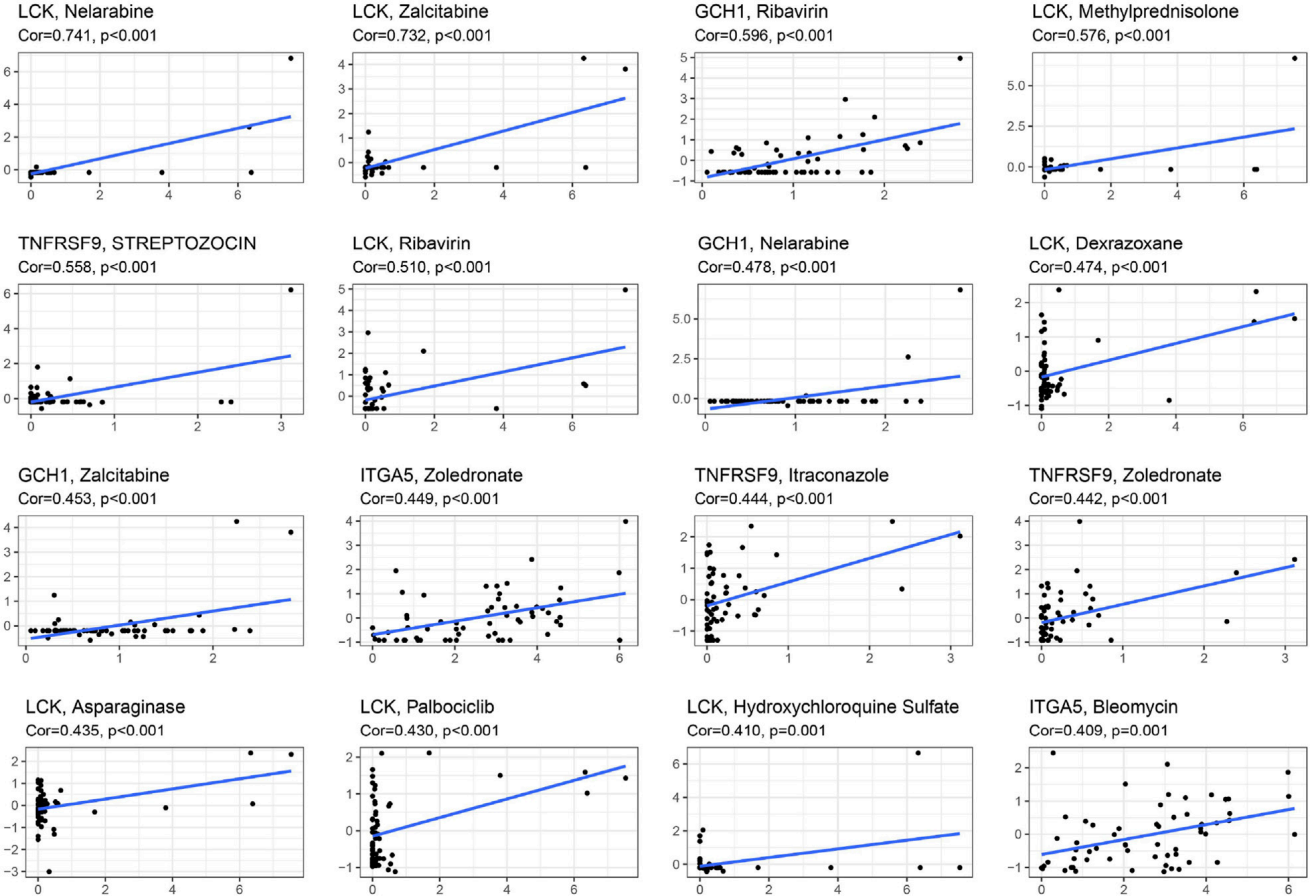
Scatter plot of relationship between prognostic gene expression and drug sensitivity by spearman correlation analysis.

In the NCI-60 cell line, we inspected the expression of prognosis-related genes and analyzed the associations between drug sensitivity and their expression levels. The findings showed that prognosis-related genes were linked to the sensitivity to some chemotherapy drugs (*p* < 0.01) (Figure 9, Supplementary Table S4). For instance, increased expression of LCK, ITGA5, CGH1 and TNFRSF9 was associated with increased drug sensitivity of cancer cells to ribavirin, nelarabine, zalcitabine, bleomycin, asparaginase, dexrazoxane, palbociclib, etc.

### 3.9 Identification of the differences in the expression of prognosis-related genes between CC tissues and normal cervical tissues

**FIGURE 10:**
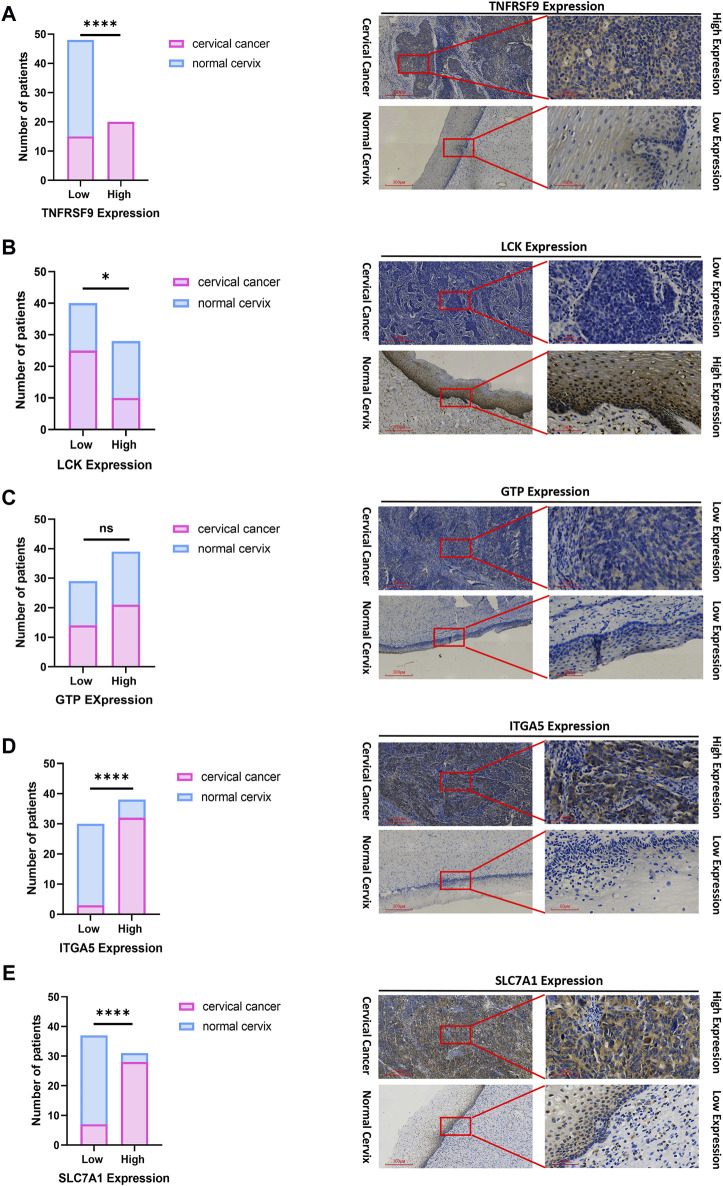
IHC confirmed the difference of the prognostic gene expression between CC and normal cervical tissues. **(A)** TNFRSF. **(B)** LCK. **(C)** GTP. **(D)** ITGA5. **(E)** SLC7A1. Representative images (×40 and ×200) of IHC staining in 35 CC and 33 normal cervical patients (high expression vs low expression). Scale bars are shown. *p < 0.05. ****p < 0.0001. ns, not significant. Values were calculated by chi-square tests.

To inspect the differences in the expression of the five prognostic genes (LCK, ITGA5, CGH1, TNFRSF9 and SLC7A1) between CC tissues and normal cervical tissues, IHC was used to analyze protein expression. IHC revealed that the prognostic genes TNFRSF9 and LCK were expressed at low levels in CC tissues (Figures 10A, B), while ITGA5 and SLC7A1 were highly expressed in CC tissues (Figures 10D, E, *p* < 0.0001). The expression of GTP was not significantly different between CC tissues and normal cervical tissues (Figure 10C).

## 4 Discussion

With the advent of gene sequencing and targeted therapies, targeted drugs for CC (other than surgery) have emerged. Because there are few validated biomarkers, we are often unable to predict treatment effects for CC. Previous studies have shown that CC biomarkers, including exosomes (Ran et al., 2022), microRNAs (Laengsri et al., 2018), and DNA and RNA molecules (Volkova et al., 2021) are associated with CC diagnosis, therapy, and prognosis. Nevertheless, the lack of reported evidence indicates that the inflammatory response-related gene signature has not been established as a prognostic marker for CC. Previous studies have suggested that the m6A-related gene signature, ferroptosis-related gene signature, immune-related gene signature and hypoxia-related gene signature predict 3-year OS for CC patients with AUCs of 0.702, 0.734, 0.785 and 0.716, respectively (Yang et al., 2021; Lu et al., 2022; Qin et al., 2022; Wang et al., 2022), which are similar to our findings. Apart from achieving high accuracy in predicting CC prognosis, the inflammatory response-related gene signature developed in our study possesses additional benefits in comparison to the aforementioned gene signatures. As an illustration, tumor drug resistance genes and immune checkpoint genes can be classified into two distinct groups based on high or low expression levels. Furthermore, the risk score derived from this signature has been determined to be significantly correlated with resistance to several chemotherapeutic drugs.

Our research focused on analyzing the expression of 200 genes associated with the inflammatory response in CC tissues and their correlation with overall survival (OS) using data from The Cancer Genome Atlas (TCGA) cohort. After screening, 63 DEGs were identified, among which 10 DEGs were found to have a significant association with OS in univariate Cox analysis. To construct a prognostic model, we used LASSO regression analysis to select 5 inflammatory response-related genes. This model was further validated using data from GSE44001. Patients were grouped into high- and low-risk groups based on the median risk score. Our analysis revealed that patients in the high-risk group had a more advanced TNM stage and shorter OS. Through both univariate and multivariate analyses, the risk score was also found to be an independent predictor of OS.

In this study, we developed a prognostic model comprising five genes (ITGA5, LCK, GCH1, TNFRSF9 and SLC7A1) that are linked to the inflammatory response. ITGA5 and SLC7A1 were found to be overexpressed in CC tumor tissues and were associated with a worse prognosis, except for LCK, GCH1 and TNFRSF9. Integrins are transmembrane receptors that are composed of two subunits, known as alpha (α) and beta (β) subunits; they form a family of approximately 24 different subtypes of α and β (Hynes, 2002). These receptors are involved in various cellular processes, such as cell adhesion, both between cells and between cells and the extracellular matrix. Additionally, integrins play important roles in cytoskeletal organization, cell migration, proliferation, and survival through their involvement in signal transduction pathways (Schnittert et al., 2018). Numerous studies have shown that the overexpression of ITGA5 is linked to unfavorable outcomes in various types of tumors, including triple-negative breast cancer (Xiao et al., 2018), ovarian cancer (Gong et al., 2016), colorectal cancer (Yu et al., 2019) and lung cancer (Zheng et al., 2016). Zhou et al. (2022) have identified five potential biomarkers—ITGA5, TGFB1, PLAU, PLAUR, and SERPINE1—for the prognosis and diagnosis of HPV-related head and neck squamous cell carcinoma (HNSCC). Moreover, these biomarker candidates are particularly implicated in the process of epithelial-mesenchymal transition (EMT). EMT is a cancer-specific biological phenomenon characterized by the loss of cellular adhesion and polarity in epithelial cells, resulting in a transition from a stationary epithelial phenotype to a migratory and invasive mesenchymal phenotype. Therefore, ITGA5 might be involve in loss of cellular adhesion and polarity in epithelial cell. Elevated levels of ITGA5 were notably associated with increased risk concerning both OS and advanced stage among CC. The genes differentially expressed in association with ITGA5 were linked to angiogenesis, as evidenced by IHC revealing a positive correlation between ITGA5 expression and microvascular density in CC tissues (Xu et al., 2023). The above findings resemble to ours, but further *in vivo* and *in vitro* functional experiments are still needed to validate the role of ITGA5 in CC.

LCK, also referred to as lymphocyte-specific protein tyrosine kinase (Rohrs et al., 2016), belongs to the Src family of nonreceptor protein tyrosine kinases. Lck has been detected in several solid tumor types, such as colon cancer (Janikowska et al., 2018), breast cancer (Santpere et al., 2018), and lung carcinoma (Mahabeleshwar and Kundu, 2003). These findings support the hypothesis that Lck may possess oncogenic properties, suggesting its potential as a diagnostic biomarker and therapeutic target for solid tumors. Hauck et al. (2012) presented a novel T-cell immunodeficiency caused by a defect in the LCK gene, emphasizing the crucial role of Lck in the development and responses of human T cells. Furthermore, abnormalities in the TCR signaling cascade frequently led to aberrant T-cell differentiation and impaired functions, thereby posing a significant risk factor for inflammation and autoimmunity. Similarly, Lui et al. (2024) reported that a partial defect in human LCK leads to T-cell immunodeficiency accompanied by inflammation in the intestines. The induction of inflammatory cell death by CD4^+^ T cells is a mechanism by which immune-evasive tumors are controlled (Kruse et al., 2023). Li et al. (2016) identified a novel mutation in the LCK gene in a family exhibiting T-cell defects, atypical EV, virus-induced malignancy and HPV infection. This finding provides new perspectives on host defence mechanisms against HPV. Currently, there is still no research on LCK in CC. Our research revealed that LCK is classified as low risk according to the risk model. Based on research on LCK in other types of tumors, we speculate that LCK may be associated with immune evasion in the inflammation-related cervical cancer risk model. Further validation through IHC suggested that there was no significant difference in the expression of GTP between the normal cervix and CC tissues. To confirm these findings, it may be necessary to increase the sample size for verification.

TNFRSF9, also known as 4-1BB, is reportedly expressed in both mouse and human tumor-infiltrating regulatory T cells (TI-Tregs) (Buchan et al., 2018). TNFRSF9 has also been identified as a marker for activating tumor-reactive Tregs (Guo et al., 2018). However, in contrast to our expectations, the expression of TNFRSF9 was found to be greater in CC tumor tissues than in myoma patient cervix (nontumorous) tissues, as validated by IHC. This finding seems to be consistent with the notion that high expression of TNFRSF9 in cancer indicates poor survival. The explanation for this discrepancy is that all the samples used for research were from non-Asian individuals in the TCGA study, while in our IHC validation, all our samples were from Asian individuals. This difference in sample populations may lead to variations in the expression and mechanisms of TNFRSF9 in different racial groups of CC patients. However, the specific mechanisms underlying its unique actions remain to be elucidated.

GCH1, the initial enzyme in the *de novo* BH4 synthesis pathway, is recognized for its expression in activated T cells (Chen et al., 2011). There are reports of GCH1 exhibiting high expression in some tumors, such as hepatocellular carcinoma (Zhong et al., 2021), esophageal squamous cell carcinoma (Gao et al., 2016), and gastric cancer (Liu et al., 2022), and its association with tumor progression, metastasis, and poor prognosis. Contrary to the previously mentioned findings suggesting that GCH1 is an oncogene, Zhong et al. (2021) proposed that GCH1 may actually inhibit the proliferation of HCC cells by promoting the accumulation of intracellular BH4. Our findings indicated that GCH1 is similarly downregulated in tumor tissues. These observations highlight the fact that the effects of GCH1 on tumorigenesis can vary in different types of cancer. But there have been no reports on the research of CGH1 in CC. Therefore, further studies are necessary to elucidate the role of GCH1 in other cancer types to gain a better understanding of its significance in cancer.

Solute carrier family 7 (SLC7) refers to a collection of membrane channel proteins that are categorized into two families, namely, L-type amino acid transporters (LATs) and cationic amino acid transporters (CATs). Recent investigations have demonstrated the association of SLC7A1 with tumor development in breast, epithelial ovarian and liver cancer (Abdelmagid et al., 2011; He et al., 2020; You et al., 2022). In hepatoblastoma (HB), SLC7A1, acting as a substrate of the tumor suppressor gene SPOP, modulates the progression of HB through the regulation of arginine metabolism, thereby offering a novel therapeutic target for HB (He et al., 2020). Our research demonstrated that SLC7A1 is highly expressed in the risk model of CC and is associated with prognosis in cervical cancer patients. The research on SLC7A1 in CC has yet to be reported. Therefore, further studies are necessary to validate the role of SCL7A1 in CC to gain a better understanding of its significance.

To further elucidate the relationship between immune components and the risk score, we examined the effect of the risk score on immune infiltration patterns. Notably, we found a significant correlation between high-risk score and C1, while a low-risk score was strongly associated with C4, suggesting that C1 promotes tumorigenesis and progression, while C4 serves as a protective factor. This discovery is consistent with previous studies, which have shown that high cytotoxicity can inhibit tumor growth and progression (Tamborero et al., 2018b). Additionally, we observed that high risk score was strongly associated with stage III-IV tumors, indicating a clear relationship between a high risk score and poor prognosis.

Nevertheless, the impact of these genes on the prognosis of CC patients through the inflammatory response remains unclear due to limited research on these genes. GSEA revealed significant enrichment of tumor-related signaling pathways, including ECM receptor interaction, Focal adhesion, and Glycosaminoglycan Biosynthesis Chondroitin Sulfate (Lin et al., 2022; Zhang et al., 2022; Sun et al., 2023). ECM receptor interactions and focal adhesion have been linked to CC and may serve as potential therapeutic targets (Chen et al., 2023; Xu et al., 2023).

Immunotherapies for cancer that target immune checkpoints, such as anti-PD-1/L1 antibodies and CTLA4, have demonstrated clinical efficacy across multiple cancer types (Pardoll, 2012). However, a significant number of cancer patients still do not respond to checkpoint inhibition therapies, with response rates ranging from 15% to 50% (Wu et al., 2021), which can be attributed to the complex nature of tumors, which can be classified into four subtypes based on the immune score: immune hot, altered-excluded, altered-immunosuppressed and immune cold (Galon and Bruni, 2019). Solid tumors are often considered “cold tumors,” characterized by a low immunoscore, limited immunogenicity, and adjuvanticity, resulting in insufficient T-cell infiltration. Moreover, immunosuppression in the tumor microenvironment (TME) poses significant challenges to effective therapy (Galon and Bruni, 2019). In our research, we observed that the expression levels of PD-1 and PD-L1 were lower in the high-risk group than in the high-risk group. Moreover, the risk score was negatively associated with the expression of PD-1 and PD-L1; this indicates that the prognostic model developed in our study can predict low expression levels of immune checkpoint molecules. Our study also revealed decreased activity of Th1 cells, Th2 cells, Thf cells, and Treg cells; T-cell costimulation; and T-cell coinhibition in the high-risk group. These findings indicate that this risk model is associated with immune escape, potentially leading to attenuation of antitumor immunity. Furthermore, the prognostic risk model also revealed whether immunotherapy should be added to the CC. Therefore, there is an urgent need for novel immunotherapeutic strategies to effectively treat cancer and immune cold-induced tumors.

In this study, the inflammatory response genes (ITGA5, LCK, GCH1, TNFRSF9 and SLC7A1) play different role in immune tumor microenvironment. Targeting ITGA5 in fibroblasts through alternation of extracellular matrix (ECM) deposition can improving colorectal cancer response to PD-L1 blockade (Lu et al., 2023). In glioma, ITGA5 was implicated in immune-related processes, distinct typical genomic alterations and key oncogenic pathways. Remarkably, ITGA5 was observed to influence the immune cell infiltration and immune microenvironment in gliomas, with higher levels of immune cell infiltration associated with elevated ITGA5 expression (Li et al., 2022). SLC7A1-mediated arginine uptake as a potential therapeutic vulnerability in non-small cell lung cancer (Gai et al., 2024). LCK regulates the initiation of T-cell development, T-cell homeostasis and TCR signaling. LCK activity can improve the efficacy of chimeric antigen receptors (CARs) and to potentiate T-cell responses in cancer immunotherapy (Bommhardt et al., 2019). LCK expression in the nucleus is induced and activated by DNA damage. Disruption of LCK expression prevents the stabilisation of RAD51, BRCA1 and BRCA2 protein expression, thereby inhibiting HR-mediated DNA repair, including inhibition of RAD51 foci formation and enhancement of γH2AX foci formation. In contrast, LCK overexpression leads to an increase in RAD51 and BRCA1 expression and an increase in HR DNA damage repair (Dey et al., 2023). CD137 (TNFRSF9, 4-1BB) modulates the infiltration of exhausted CD8^+^ T cells (Tex) in tumors expressing PD1, Lag-3, and Tim-3 inhibitory receptors. Through Tox-dependent chromatin remodeling and the RelA and cRel canonical NF-κB subunits, TCR-independent CD137 signaling promotes the proliferation and terminal differentiation of Tex precursor cells. Additionally, incorporating 4-1BB as a costimulatory domain in chimeric antigen receptor T (CAR-T) cells enhances T cell proliferation and survival while mitigating T cell exhaustion (Pichler et al., 2023). With ligand binding by 4-1BB, the nuclear factor-kappa B signaling pathway is activated, which results in transcription of corresponding genes such as interleukin-2 and interferon-γ, as well as the induction of T cell proliferation and antiapoptotic signals (Shen et al., 2023). GTP controls the function of Rac1, a guanine nucleotide-binding protein, leading to the dephosphorylation of serine 323 on Abl-interactor 1 (Abi-1) by protein phosphatase 5 (PP5). The dephosphorylated Abi-1, a protein with no previous known role in DNA repair activation, promotes nonhomologous end joining (Zhou et al., 2024). However, further research is needed to investigate the immunomodulatory role of these inflammatory genes in the tumor microenvironment of CC.

Based on the NCI-60 cell line data, we observed that the upregulation of certain prognostic genes is linked to increased sensitivity to several FDA-approved chemotherapeutic drugs, such as nelarabine, zalcitabine, bleomycin, dexrazoxane and palbociclib. The MRP family comprises 13 members, among which MRP1 to MRP9 are the primary transporters responsible for multidrug resistance, as they actively eliminate anticancer drugs from cancer cells (Chen and Tiwari, 2011). Consequently, the observed association between the risk score and the expression of drug resistance genes, such as MRP1 and MRP2, suggests that targeting drug resistance genes in tumors could be a viable therapeutic approach for high-risk patients. These discoveries emphasize the potential of harnessing specific prognostic genes as therapeutic targets, aiming to overcome drug resistance or augment drug responsiveness.

## 5 Conclusion

In summary, in this study we established a novel prognostic model comprising five genes associated with the inflammatory response. This signature demonstrated independent prognostic value for overall survival in both the TCGA and GSE44001 validation cohorts. Additionally, functional analysis, analysis of the tumor microenvironment, and determination of drug sensitivity were applied to further support the validity of the model. These findings offer valuable insights into prognostic prediction for CC. However, the specific underlying mechanisms linking inflammatory response-related genes and tumor immunity in CC remain unclear and necessitate further investigation. Overall, our research significantly contributes to the understanding of the roles of these genes in tumor development, particularly in relation to immune escape, the immune response, the tumor microenvironment, and drug resistance. This knowledge is crucial for the advancement of personalized therapies.

## Data Availability

The datasets presented in this study can be found in online repositories. The names of the repository/repositories and accession number(s) can be found in the article/Supplementary Material.

## Supplementary material

SUPPLEMENTARY TABLE S1 200 inflammatory response-related genes from the Molecular Signatures database

SUPPLEMENTARY TABLE S2 The efficacy of 314 drugs approved by the FDA.

SUPPLEMENTARY TABLE S3 Sixty-three genes were differentially expressed between tumor and paracancerous tissues.

SUPPLEMENTARY TABLE S4 Five prognosis-related genes were linked to the sensitivity to some chemotherapy drugs.

## References

[B1] Abdelmagid S. A. Rickard J. A. McDonald W. J. Thomas L. N. Too C. K. (2011). CAT-1-mediated arginine uptake and regulation of nitric oxide synthases for the survival of human breast cancer cell lines. J. Cell. Biochem. 112 (4), 1084–1092. 10.1002/jcb.23022 21308737

[B2] Armstrong H. Bording-Jorgensen M. Dijk S. Wine E. (2018). The complex interplay between chronic inflammation, the microbiome, and cancer: understanding disease progression and what we can do to prevent it. Cancers 10 (3), 83. 10.3390/cancers10030083 29558443 PMC5876658

[B3] Bommhardt U. Schraven B. Simeoni L. (2019). Beyond TCR signaling: emerging functions of lck in cancer and immunotherapy. Int. J. Mol. Sci. 20 (14), 3500. 10.3390/ijms20143500 31315298 PMC6679228

[B4] Buchan S. L. Dou L. Remer M. Booth S. G. Dunn S. N. Lai C. (2018). Antibodies to costimulatory receptor 4-1BB enhance anti-tumor immunity via T regulatory cell depletion and promotion of CD8 T cell effector function. Immunity 49 (5), 958–970. 10.1016/j.immuni.2018.09.014 30446386

[B5] Chen H. Wang J. Yang H. Chen D. Li P. (2016). Association between FOXM1 and hedgehog signaling pathway in human cervical carcinoma by tissue microarray analysis. Oncol. Lett. 12 (4), 2664–2673. 10.3892/ol.2016.4932 27698840 PMC5038455

[B6] Chen T. Wang J. Li M. Wu Q. Cui S. (2023). Genistein inhibits proliferation and metastasis in human cervical cancer cells through the focal adhesion kinase signaling pathway: a network pharmacology-based *in vitro* study in HeLa cells. Molecules 28 (4), 1919. 10.3390/molecules28041919 36838908 PMC9963694

[B7] Chen W. Li L. Brod T. Saeed O. Thabet S. Jansen T. (2011). Role of increased guanosine triphosphate cyclohydrolase-1 expression and tetrahydrobiopterin levels upon T cell activation. J. Biol. Chem. 286 (16), 13846–13851. 10.1074/jbc.M110.191023 21343293 PMC3077585

[B8] Chen Z. S. Tiwari A. K. (2011). Multidrug resistance proteins (MRPs/ABCCs) in cancer chemotherapy and genetic diseases. FEBS J. 278 (18), 3226–3245. 10.1111/j.1742-4658.2011.08235.x 21740521 PMC3168698

[B9] Cohen P. A. Jhingran A. Oaknin A. Denny L. (2019). Cervical cancer. Lancet 393 (10167), 169–182. 10.1016/s0140-6736(18)32470-x 30638582

[B10] Dey G. Bharti R. Braley C. Alluri R. Esakov E. Crean-Tate K. (2023). LCK facilitates DNA damage repair by stabilizing RAD51 and BRCA1 in the nucleus of chemoresistant ovarian cancer. J. ovarian Res. 16 (1), 122. 10.1186/s13048-023-01194-2 37370140 PMC10294509

[B11] Franciosi M. L. M. do Carmo T. I. T. Zanini D. Cardoso A. M. (2022). Inflammatory profile in cervical cancer: influence of purinergic signaling and possible therapeutic targets. Inflamm. Res. 71 (5-6), 555–564. 10.1007/s00011-022-01560-8 35376994

[B12] Gai X. Liu Y. Lan X. Chen L. Yuan T. Xu J. (2024). Oncogenic KRAS induces arginine auxotrophy and confers a therapeutic vulnerability to SLC7A1 inhibition in non-small cell lung cancer. Cancer Res. 10.1158/0008-5472.Can-23-2095 38502865

[B13] Galon J. Bruni D. (2019). Approaches to treat immune hot, altered and cold tumours with combination immunotherapies. Nat. Rev. Drug Discov. 18 (3), 197–218. 10.1038/s41573-018-0007-y 30610226

[B14] Gao Y. Wang W. Cao J. Wang F. Geng Y. Cao J. (2016). Upregulation of AUF1 is involved in the proliferation of esophageal squamous cell carcinoma through GCH1. Int. J. Oncol. 49 (5), 2001–2010. 10.3892/ijo.2016.3713 27826622

[B15] Gong C. Yang Z. Wu F. Han L. Liu Y. Gong W. (2016). miR-17 inhibits ovarian cancer cell peritoneal metastasis by targeting ITGA5 and ITGB1. Oncol. Rep. 36 (4), 2177–2183. 10.3892/or.2016.4985 27499367

[B16] Greten F. R. Grivennikov S. I. (2019). Inflammation and cancer: triggers, mechanisms, and consequences. Immunity 51 (1), 27–41. 10.1016/j.immuni.2019.06.025 31315034 PMC6831096

[B17] Guo X. Zhang Y. Zheng L. Zheng C. Song J. Zhang Q. (2018). Global characterization of T cells in non-small-cell lung cancer by single-cell sequencing. Nat. Med. 24 (7), 978–985. 10.1038/s41591-018-0045-3 29942094

[B18] Hauck F. Randriamampita C. Martin E. Gerart S. Lambert N. Lim A. (2012). Primary T-cell immunodeficiency with immunodysregulation caused by autosomal recessive LCK deficiency. J. Allergy Clin. Immunol. 130 (5), 1144–1152. 10.1016/j.jaci.2012.07.029 22985903

[B19] He W. Zhang J. Liu B. Liu X. Liu G. Xie L. (2020). S119N mutation of the E3 ubiquitin ligase SPOP suppresses SLC7A1 degradation to regulate hepatoblastoma progression. Mol. Ther. oncolytics 19, 149–162. 10.1016/j.omto.2020.09.008 33209975 PMC7644817

[B20] Hynes R. O. (2002). Integrins: bidirectional, allosteric signaling machines. Cell 110, 673–687. 10.1016/s0092-8674(02)00971-6 12297042

[B21] Ilhan Z. E. Łaniewski P. Thomas N. Roe D. J. Chase D. M. Herbst-Kralovetz M. M. (2019). Deciphering the complex interplay between microbiota, HPV, inflammation and cancer through cervicovaginal metabolic profiling. EBioMedicine 44, 675–690. 10.1016/j.ebiom.2019.04.028 31027917 PMC6604110

[B22] Janikowska G. Janikowski T. Pyka-Pająk A. Mazurek U. Janikowski M. Gonciarz M. (2018). Potential biomarkers for the early diagnosis of colorectal adenocarcinoma - transcriptomic analysis of four clinical stages. Cancer biomarkers Sect. A Dis. markers 22 (1), 89–99. 10.3233/cbm-170984 PMC13078447 29562499

[B23] Kruse B. Buzzai A. C. Shridhar N. Braun A. D. Gellert S. Knauth K. (2023). CD4(+) T cell-induced inflammatory cell death controls immune-evasive tumours. Nature 618 (7967), 1033–1040. 10.1038/s41586-023-06199-x 37316667 PMC10307640

[B24] Laengsri V. Kerdpin U. Plabplueng C. Treeratanapiboon L. Nuchnoi P. (2018). Cervical cancer markers: epigenetics and microRNAs. Lab. Med. 49 (2), 97–111. 10.1093/labmed/lmx080 29378033

[B25] Li S. Zhang N. Liu S. Zhang H. Liu J. Qi Y. (2022). ITGA5 is a novel oncogenic biomarker and correlates with tumor immune microenvironment in gliomas. Front. Oncol. 12, 844144. 10.3389/fonc.2022.844144 35371978 PMC8971292

[B26] Li S. L. Duo L. N. Wang H. J. Dai W. Zhou E. H. Xu Y. N. (2016). Identification of LCK mutation in a family with atypical epidermodysplasia verruciformis with T-cell defects and virus-induced squamous cell carcinoma. Br. J. dermatology 175 (6), 1204–1209. 10.1111/bjd.14679 27087313

[B27] Li X. Zheng R. Li X. Shan H. Wu Q. Wang Y. (2017). Trends of incidence rate and age at diagnosis for cervical cancer in China, from 2000 to 2014. Chin. J. Cancer Res. 29 (6), 477–486. 10.21147/j.issn.1000-9604.2017.06.02 29353970 PMC5758729

[B28] Lin X. Zhuang S. Chen X. Du J. Zhong L. Ding J. (2022). lncRNA ITGB8-AS1 functions as a ceRNA to promote colorectal cancer growth and migration through integrin-mediated focal adhesion signaling. Mol. Ther. J. Am. Soc. Gene Ther. 30 (2), 688–702. 10.1016/j.ymthe.2021.08.011 PMC8821934 34371180

[B29] Liu Y. Zhai E. Chen J. Qian Y. Zhao R. Ma Y. (2022). m(6) A-mediated regulation of PBX1-GCH1 axis promotes gastric cancer proliferation and metastasis by elevating tetrahydrobiopterin levels. Cancer Commun. Lond. Engl. 42 (4), 327–344. 10.1002/cac2.12281 PMC9017753 35261206

[B30] Lu L. Gao Y. Huang D. Liu H. Yin D. Li M. (2023). Targeting integrin α5 in fibroblasts potentiates colorectal cancer response to PD-L1 blockade by affecting extracellular-matrix deposition. J. Immunother. Cancer 11 (12), e007447. 10.1136/jitc-2023-007447 38040421 PMC10693881

[B31] Lu X. Li R. Ying Y. Zhang W. Wang W. (2022). Gene signatures, immune infiltration, and drug sensitivity based on a comprehensive analysis of m6a RNA methylation regulators in cervical cancer. J. Transl. Med. 20 (1), 385. 10.1186/s12967-022-03600-7 36058934 PMC9441061

[B32] Lui V. G. Hoenig M. Cabrera-Martinez B. Baxter R. M. Garcia-Perez J. E. Bailey O. (2024). A partial human LCK defect causes a T cell immunodeficiency with intestinal inflammation. J. Exp. Med. 221 (1), e20230927. 10.1084/jem.20230927 37962568 PMC10644909

[B33] Mahabeleshwar G. H. Kundu G. C. (2003). Tyrosine kinase p56lck regulates cell motility and nuclear factor kappaB-mediated secretion of urokinase type plasminogen activator through tyrosine phosphorylation of IkappaBalpha following hypoxia/reoxygenation. J. Biol. Chem. 278 (52), 52598–52612. 10.1074/jbc.M308941200 14534291

[B34] Malta T. M. Sokolov A. Gentles A. J. Burzykowski T. Poisson L. Weinstein J. N. (2018). Machine learning identifies stemness features associated with oncogenic dedifferentiation. Cell 173 (2), 338–354.e15. 10.1016/j.cell.2018.03.034 29625051 PMC5902191

[B35] Nella Prevete F. L. Amoresano A. Pucci P. de Paulis A. Rosa M. M. Melillo R. M. (2018). New perspectives in cancer: modulation of lipid metabolism and inflammation resolution. Pharmacol. Res. 128, 80–87. 10.1016/j.phrs.2017.09.024 28986132

[B36] Pardoll D. M. (2012). The blockade of immune checkpoints in cancer immunotherapy. Nat. Rev. Cancer 12 (4), 252–264. 10.1038/nrc3239 22437870 PMC4856023

[B37] Pichler A. C. Carrié N. Cuisinier M. Ghazali S. Voisin A. Axisa P. P. (2023). TCR-independent CD137 (4-1BB) signaling promotes CD8(+)-exhausted T cell proliferation and terminal differentiation. Immunity 56 (7), 1631–1648.e10. 10.1016/j.immuni.2023.06.007 37392737 PMC10649891

[B38] Qin W. He C. Jiang D. Gao Y. Chen Y. Su M. (2022). Systematic construction and validation of a novel ferroptosis-related gene model for predicting prognosis in cervical cancer. J. Immunol. Res. 2022, 2148215. 10.1155/2022/2148215 35935576 PMC9352469

[B39] Ran Z. Wu S. Ma Z. Chen X. Liu J. Yang J. (2022). Advances in exosome biomarkers for cervical cancer. Cancer Med. 11 (24), 4966–4978. 10.1002/cam4.4828 35578572 PMC9761094

[B40] Rohrs J. A. Wang P. Finley S. D. (2016). Predictive model of lymphocyte-specific protein tyrosine kinase (LCK) autoregulation. Cell. Mol. Bioeng. 9, 351–367. 10.1007/s12195-016-0438-7 27547268 PMC4978775

[B41] Santpere G. Alcaráz-Sanabria A. Corrales-Sánchez V. Pandiella A. Győrffy B. Ocaña A. (2018). Transcriptome evolution from breast epithelial cells to basal-like tumors. Oncotarget 9 (1), 453–463. 10.18632/oncotarget.23065 29416627 PMC5787480

[B42] Schnittert J. Bansal R. Storm G. Prakash J. (2018). Integrins in wound healing, fibrosis and tumor stroma: high potential targets for therapeutics and drug delivery. Adv. Drug Deliv. Rev. 129, 37–53. 10.1016/j.addr.2018.01.020 29414674

[B43] Shen X. Zhang R. Nie X. Yang Y. Hua Y. Lü P. (2023). 4-1BB targeting immunotherapy: mechanism, antibodies, and chimeric antigen receptor T. Cancer biotherapy Radiopharm. 38 (7), 431–444. 10.1089/cbr.2023.0022 37433196

[B44] Simon N. Friedman J. Hastie T. Tibshirani R. (2011). Regularization paths for cox's proportional hazards model via coordinate descent. J. Stat. Softw. 39 (5), 1–13. 10.18637/jss.v039.i05 PMC4824408 27065756

[B45] Sohrab S. S. Raj R. Nagar A. Hawthorne S. Paiva-Santos A. C. Kamal M. A. (2023). Chronic inflammation’s transformation to cancer: a nanotherapeutic paradigm. Molecules 28 (11), 4413. 10.3390/molecules28114413 37298889 PMC10254455

[B46] Sun G. Zhao S. Fan Z. Wang Y. Liu H. Cao H. (2023). CHSY1 promotes CD8(+) T cell exhaustion through activation of succinate metabolism pathway leading to colorectal cancer liver metastasis based on CRISPR/Cas9 screening. J. Exp. Clin. cancer Res. CR 42 (1), 248. 10.1186/s13046-023-02803-0 37749638 PMC10519095

[B47] Sung H. Ferlay J. Siegel R. L. Laversanne M. Soerjomataram I. Jemal A. (2021). Global cancer statistics 2020: GLOBOCAN estimates of incidence and mortality worldwide for 36 cancers in 185 countries. CA A Cancer J. Clin. 71 (3), 209–249. 10.3322/caac.21660 33538338

[B48] Tamborero D. Rubio-Perez C. Muiños F. Sabarinathan R. Piulats J. M. Muntasell A. (2018a). A pan-cancer landscape of interactions between solid tumors and infiltrating immune cell populations. Clin. Cancer Res. 24 (15), 3717–3728. 10.1158/1078-0432.Ccr-17-3509 29666300

[B49] Tamborero D. Rubio-Perez C. Muiños F. Sabarinathan R. Piulats J. M. Muntasell A. (2018b). A pan-cancer landscape of interactions between solid tumors and infiltrating immune cell populations. Clin. cancer Res. 24 (15), 3717–3728. 10.1158/1078-0432.Ccr-17-3509 29666300

[B50] Volkova L. V. Pashov A. I. Omelchuk N. N. (2021). Cervical carcinoma: oncobiology and biomarkers. Int. J. Mol. Sci. 22 (22), 12571. 10.3390/ijms222212571 34830452 PMC8624663

[B51] Wang N. Nanding A. Jia X. Wang Y. Yang C. Fan J. (2022). Mining of immunological and prognostic-related biomarker for cervical cancer based on immune cell signatures. Front. Immunol. 13, 993118. 10.3389/fimmu.2022.993118 36341424 PMC9634000

[B52] Wu C. Xu J. Xie Z. Huang H. Li N. Wei X. (2021). Light-responsive hyaluronic acid nanomicelles co-loaded with an Ido inhibitor focus targeted photoimmunotherapy against "immune cold" cancer. Biomaterials Sci. 9 (23), 8019–8031. 10.1039/d1bm01409a 34718362

[B53] Xiao Y. Li Y. Tao H. Humphries B. Li A. Jiang Y. (2018). Integrin α5 down-regulation by miR-205 suppresses triple negative breast cancer stemness and metastasis by inhibiting the Src/Vav2/Rac1 pathway. Cancer Lett. 433, 199–209. 10.1016/j.canlet.2018.06.037 29964204

[B54] Xu X. Shen L. Li W. Liu X. Yang P. Cai J. (2023). ITGA5 promotes tumor angiogenesis in cervical cancer. Cancer Med. 12 (10), 11983–11999. 10.1002/cam4.5873 36999964 PMC10242342

[B55] Yang Y. Li Y. Qi R. Zhang L. (2021). Constructe a novel 5 hypoxia genes signature for cervical cancer. Cancer Cell Int. 21 (1), 345. 10.1186/s12935-021-02050-3 34217310 PMC8254931

[B56] Yoshihara K. Shahmoradgoli M. Martinez E. Vegesna R. Kim H. Torres-Garcia W. (2013). Inferring tumour purity and stromal and immune cell admixture from expression data. Nat. Commun. 4, 2612. 10.1038/ncomms3612 24113773 PMC3826632

[B57] You S. Zhu X. Yang Y. Du X. Song K. Zheng Q. (2022). SLC7A1 overexpression is involved in energy metabolism reprogramming to induce tumor progression in epithelial ovarian cancer and is associated with immune-infiltrating cells. J. Oncol. 2022, 5864826. 10.1155/2022/5864826 36131790 PMC9484923

[B58] Yu M. Chu S. Fei B. Fang X. Liu Z. (2019). O-GlcNAcylation of ITGA5 facilitates the occurrence and development of colorectal cancer. Exp. Cell Res. 382 (2), 111464. 10.1016/j.yexcr.2019.06.009 31202709

[B59] Yuan Y. Cai X. Shen F. Ma F. (2021). HPV post-infection microenvironment and cervical cancer. Cancer Lett. 497, 243–254. 10.1016/j.canlet.2020.10.034 33122098

[B60] Zhang Q. J. Li D. Z. Lin B. Y. Geng L. Yang Z. Zheng S. S. (2022). SNHG16 promotes hepatocellular carcinoma development via activating ECM receptor interaction pathway. Hepatobiliary Pancreat. Dis. Int. HBPD INT 21 (1), 41–49. 10.1016/j.hbpd.2021.09.006 34600815

[B61] Zheng W. Jiang C. Li R. (2016). Integrin and gene network analysis reveals that ITGA5 and ITGB1 are prognostic in non-small-cell lung cancer. OncoTargets Ther. 9, 2317–2327. 10.2147/ott.S91796 PMC4846067 27143927

[B62] Zhong G. C. Zhao Z. B. Cheng Y. Wang Y. B. Qiu C. Mao L. H. (2021). Epigenetic silencing of GCH1promotes hepatocellular carcinoma growth by activating superoxide anion-mediated ASK1/p38 signaling via inhibiting tetrahydrobiopterin *de novo* biosynthesis. Free Radic. Biol. Med. 168, 81–94. 10.1016/j.freeradbiomed.2021.03.025 33781891

[B63] Zhou Q. Yuan O. Cui H. Hu T. Xiao G. G. Wei J. (2022). Bioinformatic analysis identifies HPV-related tumor microenvironment remodeling prognostic biomarkers in head and neck squamous cell carcinoma. Front. Cell. Infect. Microbiol. 12, 1007950. 10.3389/fcimb.2022.1007950 36425786 PMC9679011

[B64] Zhou W. Zhao Z. Lin A. Yang J. Z. Xu J. Wilder-Romans K. (2024). GTP signaling links metabolism, DNA repair, and responses to genotoxic stress. Cancer Discov. 14 (1), 158–175. 10.1158/2159-8290.Cd-23-0437 37902550 PMC10872631

